# Generation of dual and quad-optical frequency combs in the injected radiation free mode-locked frequency-shifted feedback laser

**DOI:** 10.1007/s12200-023-00079-y

**Published:** 2023-09-15

**Authors:** Sergey N. Mantsevich, Ekaterina I. Kostyleva, Andrey N. Danilin, Vladimir S. Khorkin

**Affiliations:** 1 Physics Department, M.V. Lomonosov Moscow State University, Moscow, 119991 Russia; 2 Russian Quantum Center (RQC), Skolkovo, 143026 Russia

**Keywords:** Frequency-shifted feedback laser, Optical frequency combs, Acousto-optics, Dual-combs, Quad-combs

## Abstract

The results of an optoelectronic system—frequency-shifted feedback (FSF) laser experimental examination are presented. The considered FSF laser is seeded only with optical amplifier spontaneous emission (ASE) and operates in the mode-locked regime, whereby the output radiation is sequence of short pulses with a repetition rate determined by the delay time in its optical feedback circuit. In the frequency domain, the spectrum of such a pulse sequence is an optical frequency comb (OFC). These OFCs we call initial. We consider the possibility of tunable acousto-optic (AO) dual and quad-comb frequency spacing downconversion in the FSF laser seeded with ASE and operating in the mode-locked regime. The examined system applies a single frequency shifting loop with single AO tunable filter as the frequency shifter that is fed with several radio frequency signals simultaneously. The initial OFCs with frequency spacing of about 6.5 MHz may be obtained in the wide spectral range and their width, envelope shape and position in the optical spectrum may be tuned. The dual-combs are obtained with a pair of initial OFCs aroused by two various ultrasound waves in the acousto-optic tunable filter (AOTF). The dual-combs frequency spacing is determined by the frequency difference of the signals applied to the AOTF piezoelectric transducer and can be tuned simply. The quad-combs are obtained with three initial OFCs, forming a pair of dual-combs, appearing when three ultrasound frequencies feed the AOTF transducer. The quad-combs frequency spacing is defined by the difference between the frequency spacing of dual-combs. Quad-combs with more than 5000 spectral lines and tunable frequency spacing are observed. The successive frequency downconversion gives the possibility to reduce the OFC frequency spacing form several MHz for initial OFC to tens of kHz for quad-combs.

**Graphical abstract:**

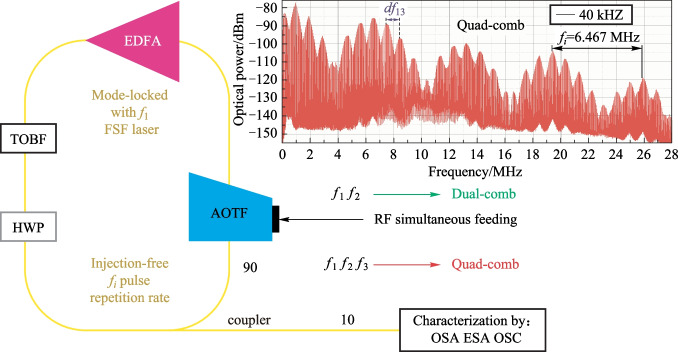

## Introduction

The development of optical frequency combs (OFCs) generation methods [1–6] and examination of their possible practical applications [7–11] is an important and intensively expanding area of optoelectronics.

To date, a number of methods have been proposed to obtain OFCs with different characteristics in various bands of the optical radiation spectrum [12]. One of the methods applies optoelectronic systems with frequency shifting loop (FSL) [6, 13]. In such devices, the OFC is implemented by successive shifting of the optical seeding signal frequency by means of a single sideband (SSB) modulator. As a rule, the role of such a modulator is performed by an electro-optic (EO) [14, 15], or much less frequently, an acousto-optic (AO) modulator [16–20].

In recent papers, it has been shown that the acousto-optic tunable filter (AOTF) can be also applied as a device responsible for shifting the frequency of light [21, 22]. The AOTF application allows control of all the OFC characteristics by changing the parameters of AO diffraction [23, 24].

The AO OFCs generation system with FSL includes an optical pump source (laser), an optical amplifier, light polarization control devices, and an AO cell [6]. The feedback circuit is closed in the presence of AO diffraction in the AO cell and can be implemented either with optical fiber or in free space and even in a thin-film design [25].

With each passage of light through the AO cell, the optical radiation frequency is shifted by the frequency of ultrasound aroused in the AO cell, due to the Doppler effect. The frequency shift can be implemented either to lower or higher frequencies, depending on the choice of − 1 or + 1 diffraction order.

It is important to state here that an almost identical optoelectronic system is also called the frequency-shifted feedback (FSF) laser [26–30]. And if, despite the presence of certain advantages over EO modulators, the AO devices are rarely used to obtain OFCs in FSL scheme, then the application of AO devices in FSF lasers is rather the rule.

FSF lasers possess a number of interesting properties. So, in the presence of an optical resonator, their radiation does not contain optical modes and is sufficiently broadband [27–33]. Therefore, they also received the name of “modeless lasers”. FSF laser radiation spectral range can be tuned over a fairly wide band by changing the parameters of the feedback circuit [32, 34–36]. In addition, with a certain selection of operating parameters, despite the optical mode’s absence, it is possible to implement an operation mode similar to the mode-locking regime in conventional lasers, which, by analogy, is called the mode-locking regime [32, 35–39]. In this case, the FSF laser output signal represents the sequence of short pulses with a repetition rate determined by the time delay in the feedback loop [40]. In frequency domain, such signal is an optical frequency comb (OFC). We will call it further the initial OFC.

Previous studies have shown that despite certain features of FSF lasers operation, they are able to generate pico- and femto-second pulses with characteristics suitable for various practical applications: high-resolution distance ranging [31, 41–43], atomic cooling and slowing [44], efficient optical pumping of atoms [45], dispersion measurement [46].

Another feature of FSF lasers is that they are able to operate without an external optical radiation injection [33, 43, 47], due only to the presence of optical amplifier spontaneous emission (ASE). In this case, their optical radiation characteristics also turn out to satisfy the requirements for a large number of practical purposes [43].

Interestingly, despite the fact that a sequence of short pulses in the spectral representation gives a discrete set of spectral lines—an optical frequency comb [39, 48–50], in the field of FSF lasers, little attention is paid to the OFCs generation abilities.

In this paper, we examine the features of OFCs generation in FSF laser operating in the mode-locked regime in the absence of external optical seeding. This means that the considered system is much simpler and cheaper than the known systems for OFCs generation, since they usually include expensive, highly stable lasers with narrow emission lines.

It is shown that the mode-locking regime can be observed in a certain frequency range of ultrasound excited in the AO cell. This study proposes for the first time to generate dual- and quad-optical frequency combs with FSF laser. All combs are obtained with single AO cell, which is fed with several RF signals simultaneously.

It was found that, despite the fact that initial OFCs in such a system had the line spacing that did not depend on the ultrasound frequency in the AO cell. For dual- and quad-combs, the spectral interval was determined by the frequency difference of the RF signals feeding to the AO cell piezoelectric transducer. Thus, the frequency spacing could be easily tuned in a broad band. Quad-combs with more than 5000 spectral lines and tunable frequency spacing were obtained. The successive dual- and quad-comb frequency downconversion gives the possibility to reduce the OFC frequency spacing from several MHz for initial OFCs to tens of kHz for quad-combs.

## Experimental setup

**Fig. 1:**
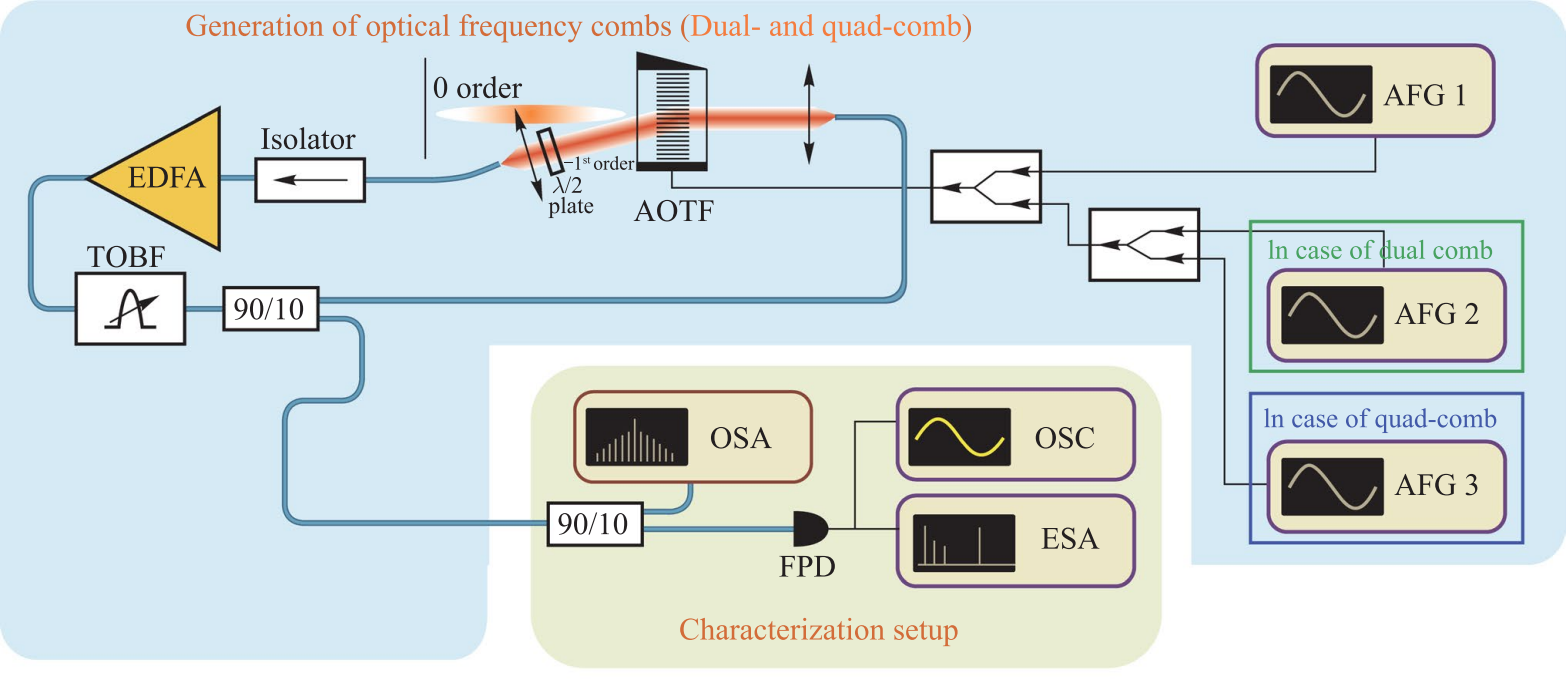
Experimental setup

The experimental setup scheme is shown in Fig. 1. It is typical for FSF lasers but includes several distinguishing features. The first feature is that paratellurite (TeO_2_) AO cell (AOTF not AOM) with 8° cut angle (angle between [110] crystallographic axis and acoustic wave vector) that plays the role of SSB modulator is a key element of FSL [38]. AOTF applies wide-angle AO diffraction geometry [51]. The AO phase-matching frequency for 1.55 µm optical radiation equals 34.5 MHz, and AOTF optical bandwidth near 1.55 µm is 36 nm.

The second feature of the setup is that it is seeded only with the spontaneous emission of optical amplifier [33, 43, 47], which is a polarization-maintaining (PM), Er/Yb double-clad silica fiber amplifier with diode laser (910–980 nm) pump source and with 33 dBm saturated output power. The input optical signal is first amplified in a low-noise preamplifier and then boosted in a power amplifier.

A PM 90/10 1 × 2 coupler ((1550 ± 15) nm passband) is used to extract optical power out of the loop and send light to the characterization setup. Characterization setup also contains 90/10 1 × 2 coupler, optical spectrum analyzer (OSA) and photodetector (FPD) connected to the electrical signal spectrum analyzer (ESA) and oscilloscope (OSC) through a broadband amplifier. The bandwidth of the characterization setup electrical part is limited by the upper frequency of the amplifier, which is about 18 GHz. The amplifier lower frequency is about 50 kHz.

The FSL, starting from coupler output, contains a PM optical fiber isolator and fiber collimator to couple light out of the fiber. Laser beam diffracts in the AOTF with more than 80% diffraction efficiency if needed. The − 1st AO diffraction order is used, thus the optical frequency is shifted down on the ultrasound frequency. It was shown in Ref. [23] that the application of the − 1st diffraction order gives the possibility to obtain broad OFCs.

The diffracted light passes through the half-wave plate (HWP) to make the polarization of diffracted light the same as the incident one has. After the HWP, the optical beam is coupled to the PM fiber again and amplified. After the amplifier, light passes through the pair of tunable (1535–1565 nm) optical 2 nm bandpass filters (TOBF) that are applied for controlling the system optical passband width and position in the spectrum.

The third distinguishing feature is the application of several arbitrary function generators (AFG1-3) feeding the same AOTF simultaneously as AO cell transducer supports multiple input RF frequencies. This means that several ultrasonic waves with different frequencies will exist in the AO cell together, and optical radiation will be diffracted by them independently. In this case, the light will obtain various frequency shifts equal to the frequencies of the acoustic waves. So multiple OFCs may be generated in the same FSL with single AOTF [24].

The single generator (AFG1) is used to obtain an initial OFC when seeding the system with ASE. The pair of generators (AFG1 and AFG2) (or single with two independent outputs) is needed to get dual-comb. Three generators (AFG1, AFG2 and AFG3) are applied to obtain quad-comb.

## Experimental results

The following experiments were carried out: examination of the ASE spectrum for various parameters of optical amplifier operation; the study of initial OFCs generated in the system in the mode-locked operation regime and their dependence on RF signal frequency applied to the AOTF; generation and examination of dual-combs; investigation of quad-combs generation.

### Optical amplifier spontaneous emission and initial OFCs generation

It was mentioned above that the only optical radiation source in the system was the optical amplifier and its spontaneous emission. So, the spectral characteristics of ASE and their dependence on the parameters of the amplifier operation were examined first. The applied optical amplifier had two tunable parameters—preamplifier current and power amplifier current. The ASE spectrum shape depended mainly on the preamplifier current. The ASE measurements were done without TOBF.

**Fig. 2:**
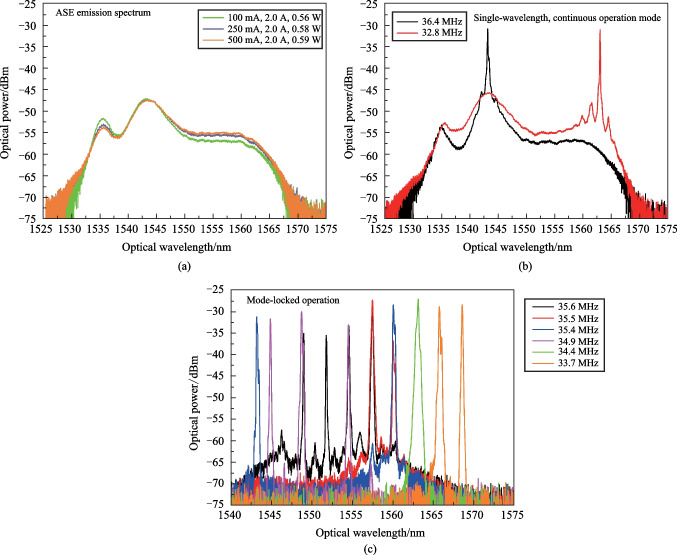
**a** Measured ASE spectra and dependence of optical amplifier preamplifier current; **b** FSF laser optical radiation spectra in the single-wavelength continuous operation mode; **c** FSF laser optical radiation spectra in the mode-locked pulsed operation regime

The spectral dependencies of ASE optical power were obtained for variation of amplifier preamplifier current from 100 to 500 mA (the maximal possible value). These results are shown in Fig. 2a. The ASE spectral band from 1530 to 1570 nm was limited by the setup optical elements spectral transmission. The shape of the spectra depended on current in a rather complicated way, but preamplifier current magnitude did not have great impact. In the short-wavelength range (up to 1545 nm), the optical emission power decreased with increasing current, and in the long-wavelength part, it increased. The further OFCs generation experiments were fulfilled for 250 mA current.

The next series of experiments were devoted to the examination of initial optical frequency combs generation. It is known that FSF lasers may operate in two regimes—the continuous-wave (CW) regime and the mode-locked regime [28, 33, 52]. If we feed the AOTF piezoelectric transducer with RF signal, in a certain frequency range (defined by the ASE spectral band) it leads to the closing of the optical feedback circuit due to the appearance of diffracted light and the system starts to operate as the FSF laser.

For the chosen AOTF the optical feedback loop is closed in the frequency band between 33 and 37 MHz. This frequency band is defined by the acousto-optic diffraction geometry in applied AOTF and corresponds to the acousto-optic diffraction phase matching frequency for the optical radiation with wavelengths near 1550 nm. The exact values of the frequency range limits are determined by a number of factors: spontaneous emission spectrum of the amplifier, its gain, the ultrasound power in the AO cell (AO diffraction efficiency), the feedback loop time delay and optical loops.

It is notable that the frequency band in which the mode-locked regime is observed lies between the bands where the system operates in the single-wavelength, continuous operation mode. The boundary between the modes is easily distinguishable by two criteria—the OFC appearance registered by ESA and a significantly different level of background radiation.

The output optical radiation spectra observed in the CW operation regime are shown in Fig. 2b. A distinctive feature of this regime is that the single band was is observed in the radiation spectrum. Its position in spectrum may be controlled by changing the ultrasound frequency in the AO cell.

The optical radiation spectra observed with OSA for the mode-locking regime are shown in Fig. 2c. Since the OSA has a spectral resolution significantly worse than the spectral interval between the OFC individual lines, each band presented in Fig. 2c is the OFC envelope. In the mode-locked regime, the OFCs exist in general case in several equidistant spectral bands [33] (up to six bands were observed in the experiment with approximately 2.8 nm spacing). The variation of the ultrasound power and the optical amplifier gain lead to a change in the envelope shape and width of the bands, while their position does not change. The system time delay (the length of the feedback circuit) variation leads to a change in the bands width and shifts in their spectral position. The width of each band is about 1 nm, this value is typical for FSF lasers.

The OFCs existence was observed for the ultrasound frequency range between 33 and 35.9 MHz for the system parameters applied in the experiments.

It is known that the spacing between the FSF lasers initial OFC spectral components is inversely proportional to the feedback loop delay time [40]. This is a significant difference from the case of AO OFCs generation in systems with FSL in the presence of external optical pump with laser radiation, when the comb lines spacing equals to the ultrasound frequency aroused in the AO cell [6, 23].

For example, in the case, shown in Fig. 2c, the *f*_*i*_ frequency interval between initial OFC lines, measured by ESA equals 7.498 MHz, while in the FSL scheme, with the same AOTF and 1549.1 nm optical pump, it will be close to 34.5 MHz and can be tuned in a bandwidth of about 2 MHz [24].

**Fig. 3:**
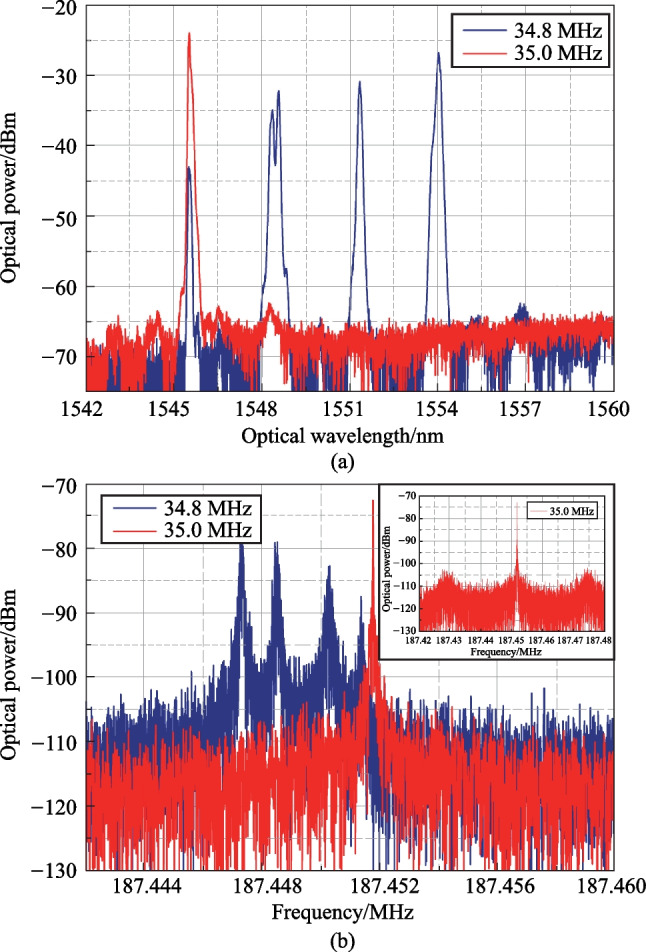
**a** OFC optical radiation spectra with 4 and 1 spectral bands; **b** corresponding OFC RF spectra single line shape, with inset showing the single peak case over a broader frequency range

The correspondence between the optical and electrical spectra in the presence of several spectral bands is illustrated by Fig. 3.

Careful examination of the OFC RF spectra with ESA (Fig. 3b) have shown that each RF spectral line in general case includes several narrow lines, the number of which equals to the number of OFC spectral bands observed in the optical spectrum (Fig. 3a). This means that each spectral band observed in Fig. 2c is to separate OFC with its own frequency spacing. The difference between the values of frequency spacing is due to the optical fiber refraction index dispersion in the examined optical wavelength range. This effect paves the way to optical fiber dispersion measurements.

The presence of several maxima within a single spectral line can be considered undesirable. Therefore, for further experiments, we applied the setup variant when the FSF laser OFC spectrum includes single optical band. This was achieved with one or a pair of mechanically tunable optical bandpass filters (TOBFs) with a 2 nm passband.

The application of single TOBF made it possible to obtain an OFC up to 2 nm wide at an ultrasound frequency of about 34.5 MHz, with a mechanically tunable position in the spectrum in the range from 1535 to 156 nm. In this case, the long-wavelength boundary was limited by the TOBF characteristics. The ultrasound frequency range, in which it was possible to obtain such combs, depended on the RF generator signal amplitude and the optical amplifier gain (the larger their values, the wider the frequency band).

A pair of TOBFs was applied to obtain narrower combs. In this case, the TOBFs transmission bands were shifted relative to each other. The TOBFs were adjusted so that the comb width was limited about 1 nm for convenience of registration. In such configuration of the optical feedback loop, the RF spectral interval *f*_*i*_ between the OFC components defined by the time delay in the feedback loop was 6.467 MHz.

**Fig. 4:**
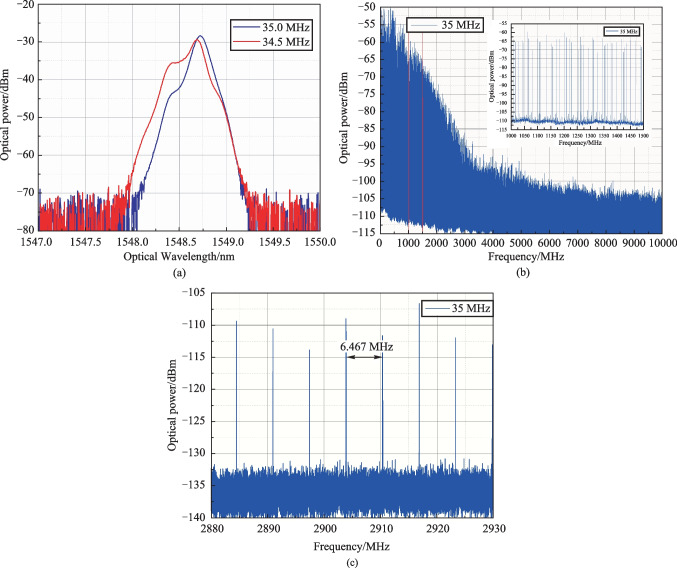
Generated OFC: **a** optical spectra for 34.4 and 35.0 MHz ultrasound frequencies; **b** OFC RF spectrum for 35 MHz ultrasound frequency; **c** OFC RF spectrum line spacing, independent on ultrasound frequency

Some of the obtained OFC optical spectra for varying AFG1 ultrasound frequency *f*_1_ are shown in Fig. 4a. Corresponding RF spectra (first 10 GHz) for *f*_1_ = 35 MHz measured by ESA are presented in Fig. 4b with 1–1.5 GHz inset. Figure 4c illustrates the comb lines spacing *f*_*i*_ that depends on the time delay in FSL.

### Dual-combs generation in FSF laser with ASE seeding

Comparatively broad spectral width of the generated initial OFCs makes the registration of their RF spectrum a difficult task. At the same time, the applied in the experimental setup electronics cut-off frequency is limited by approximately 18 GHz.

Therefore, it seems reasonable both from practical and scientific points of view to lower the RF spectrum frequency applying dual-comb downconversion technique [11, 14, 17, 19, 53]. The method to obtain dual-combs in the considered system and the results of their characteristics examination are presented in this section of the paper.

Here we apply the same method of dual-comb downconversion that was previously proposed in [24]—a scheme with a frequency shifting loop and single AOTF as the frequency shifter, but without injection of external laser radiation.

In this case, an important feature of AO devices is used—the possibility to feed the AO cell piezoelectric transducer with several independent RF signals simultaneously. So, ultrasonic waves with the corresponding frequencies and amplitudes will be excited in the AO crystal, and the light radiation will diffract by these waves independently. We need to apply a pair of generators AFG1 and AFG2 with *f*_1_ and *f*_2_ frequencies to feed AOTF and to obtain dual-comb. The magnitudes of AFG1 and AFG2 signals were chosen to be equal.

This feature makes it possible to significantly simplify the optical scheme—to use single FSL [24] instead of a pair [53], and to arrange for the optical paths of light beams to be diffracted by different ultrasonic waves identical.

The initial OFCs with *f*_*i*_ spacing generated from the ASE have such characteristics that the minimal difference in the *f*_1_ and *f*_2_ frequencies, when the dual-combs are observed, is about 300 kHz. The maximal difference corresponds to the frequency band in which initial OFCs are generated in this system—approximately 1.5 MHz.

An interesting feature is that the initial OFCs, the interference of which leads to the appearance of dual-combs, have the same spectral lines spacing *f*_*i*_, since it is determined only by the time delay in the optical feedback loop, but the frequency interval between the dual-comb lines d*f* is determined precisely by the frequency difference of the ultrasonic waves, excited in the AO cell (d*f*_12_ = *f*_1_ − *f*_2_), and can be tuned by changing AFG1 or AFG2 frequencies.

Thus, applying dual-combs, it is possible to reduce the OFC electric spectrum upper frequency in approximately 15 times relative to the frequency of the initial comb.

**Fig. 5:**
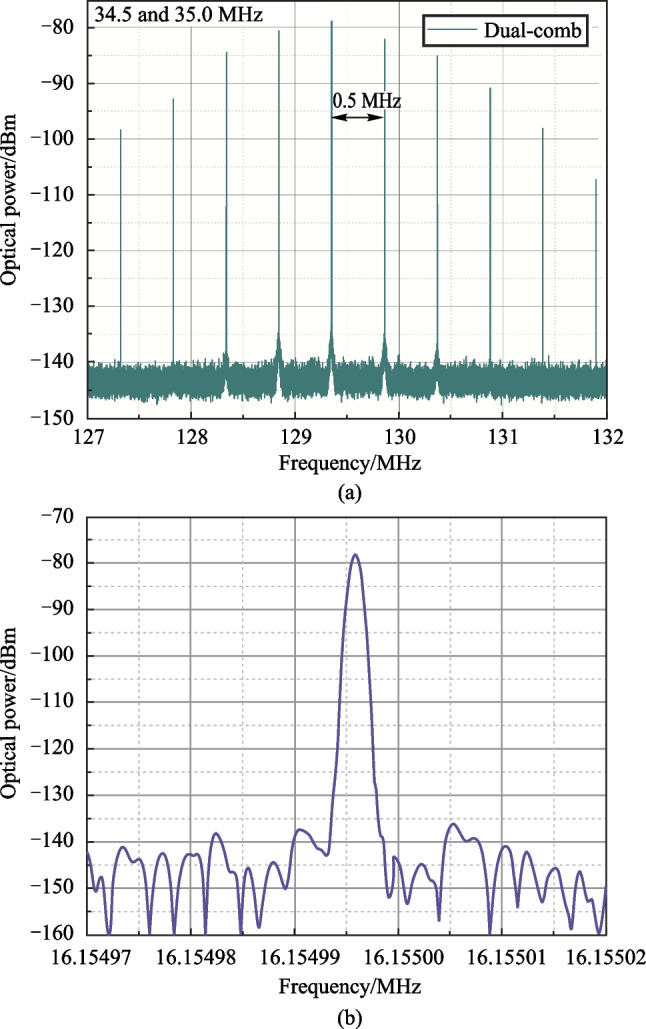
Part of the dual-comb RF spectra with **a** 0.5 MHz line spacing; **b** single dual-comb line

Part of the dual-comb RF spectrum (for *f*_1_ = 35 and *f*_2_ = 34.5 MHz frequencies) is presented in Fig. 5a. The spacing between dual-comb spectral lines is 0.5 MHz and equals the difference between two ultrasound frequencies. The single dual-comb line is shown in Fig. 5b.

**Fig. 6:**
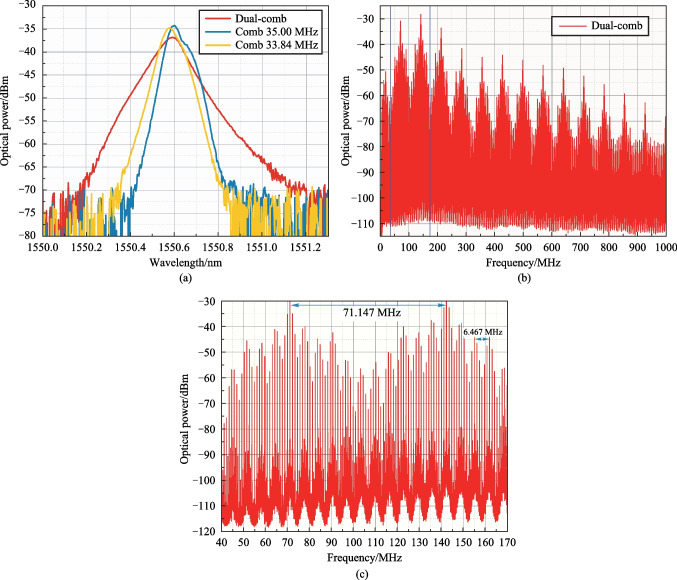
Generated dual-comb: **a** OSA spectra for initial *f*_2_ = 33.84 MHz and *f*_1_ = 35 MHz OFCs and resulting dual-comb; **b** dual-comb RF spectrum; **c** 40–170 MHz dual-comb RF spectrum

As was mentioned above, dual combs may be obtained for a fairly wide range of d*f* frequencies, ranging from about 300 kHz to approximately 1.5 MHz. Figure 6 illustrates the case when d*f*_12_ = 1.16 MHz, and with the TOBFs adjusted in such a way that the spectral width of both initial OFCs does not exceed 0.5 nm with the central wavelength near 1550.5 nm.

Figure 6a represents two initial OFCs spectra and the spectrum of the dual-comb, produced by their interference. It is possible to notice that dual-comb has a specific envelope shape, which differs from the shape of the initial combs [35, 36, 38]. The initial combs, in turn, have a radiation spectrum shape typical for the FSF laser in the case of − 1st order AO diffraction application [37, 38].

The part (first 1 GHz) of the dual-comb RF spectrum and its detailed image in the frequency band from 40 to 170 MHz are shown in Fig. 6b and c correspondingly. The dual-comb spectrum envelope, in contrast to the spectra of OFCs obtained in systems with a FSL, and from the initial OFC RF spectrum, is not smooth. It consists of a set of equidistant peaks, gradually decreasing in amplitude with frequency. For the examined system, the frequency interval between these peaks equals to 71.147 MHz, which corresponds to 11*f*_*i*_.

The detailed visualization of the dual-comb spectrum, represented in Fig. 6c, shows that main peaks consist of a set of smaller maxima, the frequency interval between which corresponds to the *f*_*i*_ frequency. Finally, all these peaks consist of the dual-comb lines, with the frequency spacing being set by the d*f*_12_ = *f*_1_ − *f*_2_.

### Quad-combs generation with ASE seeding

We showed in the previous section of the paper that it is possible to obtain dual-combs with tunable frequency interval between spectral lines in the examined system. It was also found that the minimal value of this interval was limited to a few hundred kHz.

On the one hand, such frequencies are significantly lower than the typical spectral line spacing for OFC generation systems with AO FSL. On the other hand, in the case of OFCs with spectral width of about 2 nm, such interval values still lead to the need to measure the RF signals with upper frequencies of several tens of GHz. Therefore, it is desirable to reduce the frequency spacing to about tens of kHz.

We propose to further downconversion by the interaction of a pair of dual-combs with different d*f* values. The possibility of simultaneous AO device operation with several radio signals (*f*_1_, *f*_2_ and *f*_3_) also gives such an opportunity.

**Fig. 7:**
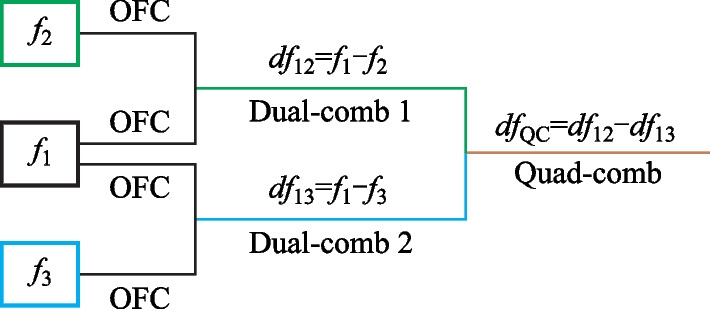
Quad-comb generation scheme

Figure 7 shows the scheme for a quad-comb [54–58] generation (we call it quad-comb as it is the result of two dual-combs interference). It is implemented in the following way. Let us connect three signal sources (AFG1-3 in Fig. 1) to the piezoelectric transducer of the AO cell. These generators should run at different frequencies *f*_1_, *f*_2_ and *f*_3_ correspondingly; if necessary, we are also able to control their amplitudes and phases. Then, for each signal in the feedback loop, its own initial OFC will be aroused (OFC in the diagram), with the same *f*_*i*_ = 6.467 MHz spectral interval, determined by the time delay in the feedback loop. These combs will interact and produce dual-combs.

To obtain single quad-comb one should choose frequencies *f*_1–3_ in the following way: *f*_1_ − *f*_2_ ≈ *f*_1_ − *f*_3_ ≫ *f*_2_ − *f*_3_ given that *f*_2_ − *f*_3_ < 300 kHz. We have also chosen *f*_1_ > *f*_2_ > *f*_3_. Then the interaction between initial OFCs form AFG2 and AFG3 will not give rise to a dual-comb, and there will be only two dual-combs with frequency intervals d*f*_12_ = *f*_1_ − *f*_2_ and d*f*_13_ = *f*_1_ − *f*_3_. The interaction of these two dual-combs makes it possible to obtain a quad-comb with a frequency spacing d*f*_QC_ equal to the frequency difference d*f*_12_ − d*f*_13_ = *f*_3_ − *f*_2_. Thus, it is possible to obtain a quad-comb with a frequency interval d*f*_QC_ varying from a few hundred kHz to tens of kHz.

**Fig. 8:**
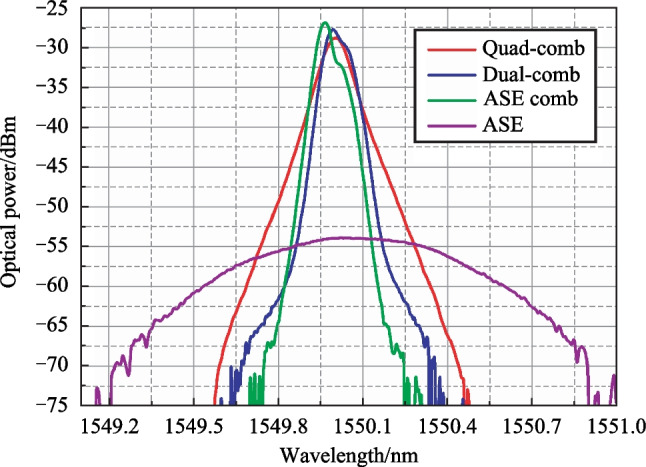
Optical spectra measured by OSA: ASE, initial ASE OFC, dual- and quad-comb optical spectra

**Fig. 9:**
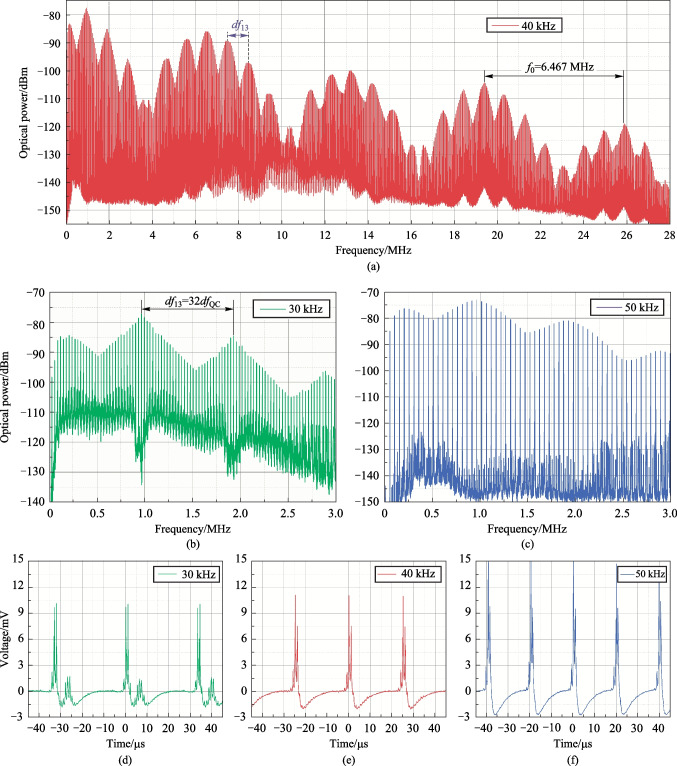
Quad-comb examination results: **a**–**c** quad-comb RF spectra for 40 (0–28 MHz band), 30 and 50 kHz (0–3 MHz band); **d**–**f** quad-combs representation in time-domain for 30, 40 and 50 kHz line spacing

**Fig. 10:**
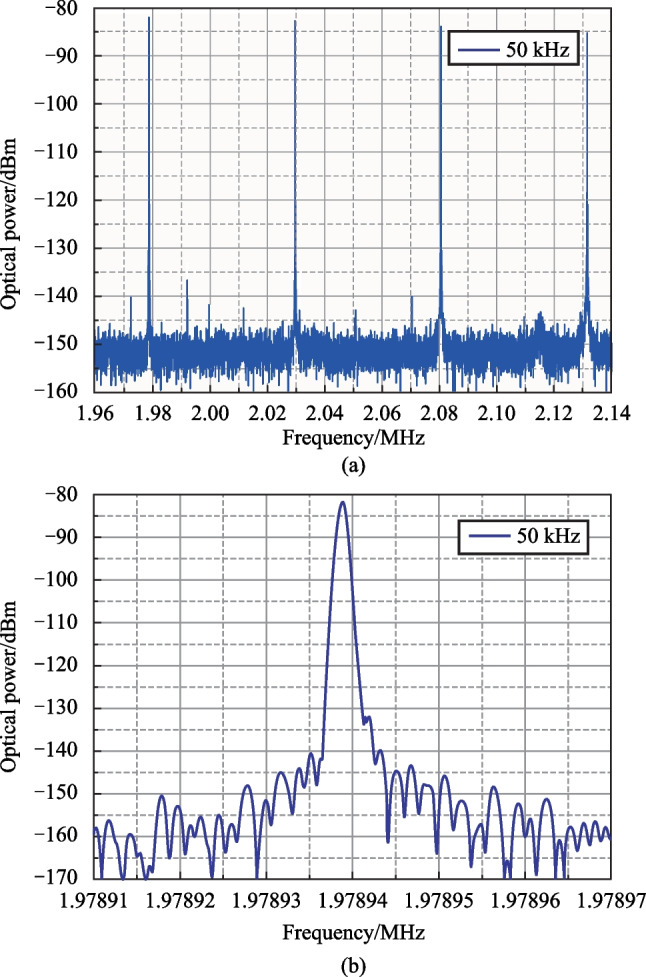
Quad-comb RF spectrum lines for 50 kHz spacing: **a** 4 lines; **b** single line

Figures 8, 9, 10 represent the results of quad-combs examination. The optical spectra of obtained combs and seeding ASE (optical system bandwidth is limited to 2 nm) are shown in Fig. 8. The ASE comb here is the initial OFC with *f*_*i*_ spacing. It can be seen that its envelope has a typical shape [35, 36]. Three such combs for *f*_1_,* f*_2_ and *f*_3_ ultrasound frequencies form two dual-combs. The dual-combs envelopes do not differ significantly, so the figure shows only one of them—dual-comb 1 with d*f*_12_ spacing.

The quad-comb envelope is symmetrical about the central wavelength, and its spectral width (0.9 nm) is higher than that for dual-combs.

The results of quad-comb RF spectra examination, represented in Fig. 9a–c (*f*_1_ = 35 MHz), show that the spectra of dual- and quad-combs differ significantly. The spectrum of the quad-comb also has a periodic structure, with a period equal to the *f*_*i*_ frequency, consisting of a set of maxima. At the same time, the maxima are equidistant with a *f*_13_ frequency interval. The spacing between the comb lines d*f*_QC_ = *f*_3_ − *f*_2_ and could be varied. In the experiment, the upper frequency of the quad-comb spectrum exceeded 200 MHz. Thus, for 40 kHz spacing (Fig. 9a illustrates first 28 MHz of the quad-comb RF spectra), the number of registered comb lines was more than 5000.

During the experimental studies, the value of d*f*_QC_ frequency was changed only by tuning the *f*_3_ frequency; therefore, the shape of the tri-comb RF spectrum for other d*f*_QC_ values shown in Fig. 9b and c (30 and 50 kHz) does not differ much from that at 40 kHz.

An important issue is that the quad-comb spectral lines with d*f*_QC_ spacing are obtained from dual-comb lines with a d*f*_13_ interval, while each dual-comb spectral component generates its own quad-comb lines. Therefore, in order for the quad-comb components obtained from different dual-comb lines to match in frequency, the d*f*_QC_ value must be a multiple of the d*f*_13_ frequency interval (d*f*_QC_ = d*f*_13_/*n*, where *n* is and integer), up to the width of the quad-comb spectral line. Otherwise, each component of comb RF spectra acquires several lateral components of smaller amplitude, equidistantly spaced by a frequency *|*d*f*_QC_ − d*f*_13_/*n|*.

Thus, in the case of 30 kHz spacing (Fig. 9b) the 32 quad-comb lines are disposed between two dual-comb lines. In the experiment d*f*_13_ = 964 kHz, so d*f*_QC_ = d*f*_13_/32 = 30.125 kHz (corresponds to 30 kHz as the nearest integer). Therefore, d*f*_QC_ frequency cannot be arbitrary, and its values form a discrete set of frequencies (d*f*_QC_ = d*f*_13_/*n*, two nearest frequencies are 31.0968 and 29.2121 kHz). The same situation takes place for other d*f*_QC_ frequencies, including 40 and 50 kHz (d*f*_13_/24 = 40.1667 and d*f*_13_/19 = 50.7368 kHz correspondingly).

The representation of quad-combs in time domain measured by oscilloscope is shown in Fig. 9c–f. It is possible to see that in the time domain, the examined AO quad-combs are the sequences of pulses, as well as dual-combs [24, 55, 56, 59]. The pulses repetition rate is determined by the d*f*_QC_ spacing. The amplitude of each pulse and the number of oscillations depend on the number of comb spectral lines.

A detailed view of the quad-comb with 50 kHz line spacing in a narrow frequency range near 2 MHz frequency and the shape of a quad-comb single line measured with a 1 Hz frequency resolution are shown in Fig. 10.

The comparison of dual-comb line (Fig. 5b) with quad-comb line (Fig. 10b) shows that their characteristics are approximately the same.

## Conclusion

We have considered various aspects of the FSF laser functioning when seeded with optical amplifier spontaneous emission and applying AOTF as the frequency shifter.

The study has shown that the examined system may operate both in the single-wavelength continuous operation mode and in the mode-locked regime, depending on the frequency of ultrasound aroused in the AOTF.

The main attention was given to the examination of the system operation peculiarities in the mode-locked regime. In this case, the output radiation in the time domain is a sequence of pulses with a repetition rate determined by the feedback loop time delay. In the spectral domain, such signal corresponds to the optical frequency comb (initial OFC).

It was found that in the general case, without additional system passband limitation with TOBFs, the OFC optical spectrum in mode-locked regime may have included up to six equidistant bands simultaneously, each with a half-width of about 1 nm. In this case, the OFC RF spectrum lines contained as many closely spaced maxima as there are spectral bands in the optical spectrum. This means that several OFCs simultaneously existed in the system with an interval between spectral components that differed slightly due to the dispersion of the optical fiber refractive index.

If the FSL passband was limited by an additional TOBF, the single spectral band (single OFC) was observed with a width equal to the TOBF passband. The position of the OFC in the spectrum could be tuned by adjusting the filter. Such a tuning could be implemented for any ultrasound frequency for which the mode-locking takes place.

We proposed to fulfill, for the first time, the initial OFCs dual- and quad-comb downconversion with the single AOTF when applying several RF signals to its piezoelectric transducer simultaneously.

The study has shown that, when generating dual-combs, this approach made it possible to obtain combs with a spectral width of at least 1 nm (artificially limited by the TOBF), and spectral components frequency spacing, determined by the difference of the applied RF signals frequencies, varying in the range from 300 kHz to 1.5 MHz. The specific limits of this range were determined by the system parameters—the optical amplifier gain, TOBFs passband adjustment, and the FSL loops. The resulting dual-combs had a specific shape—the amplitude of their RF spectrum components varied periodically. The maximum amplitudes were observed at frequencies that were multiples of the initial OFC frequency.

Simultaneous feeding of AOTF piezoelectric transducer with three RF signals made it possible to obtain a quad-comb, with an even smaller frequency spacing between the RF spectrum components. The case when the frequency interval was several tens of kHz was considered.

The quad-comb RF spectrum also had a complicated shape—it had a periodic structure with a repetition period determined by the frequency spacing between the spectral components of the initial OFC and consisted of a set of maxima. The frequency interval between these maxima was determined by the frequency difference between AFG1 and AFG3 RF signals, while the spacing between the comb lines was determined by the frequency difference of AFG2 and AFG3 signals. Quad-combs with more than 5000 lines were obtained in the experiment.

Thus, we have shown that in a FSF laser without external optical seeding (which simplifies and significantly reduces the cost of the system), it is possible to generate optical frequency combs with tunable parameters and with frequency spacing between components of several tens of kHz. The characteristics of the obtained combs are such that they can be used for practical applications such as spectroscopy and refraction coefficient measurements.

## Data Availability

Data are available by request from the authors.

## References

[1] Cundiff ST, Ye J (2003). Colloquium: femtosecond optical frequency combs. Rev. Mod. Phys..

[2] Ohara T, Takara H, Yamamoto T, Masuda H, Morioka T, Abe M, Takahashi H (2006). Over-1000-channel ultradense WDM transmission with supercontinuum multicarrier source. J. Lightwave Technol..

[3] Del’Haye P, Schliesser A, Arcizet O, Wilken T, Holzwarth R, Kippenberg TJ (2007). Optical frequency comb generation from a monolithic microresonator. Nature.

[4] Li J, Qu Y, Yu R, Wu Y (2018). Generation and control of optical frequency combs using cavity electromagnetically induced transparency. Phys. Rev. A (Coll. Park).

[5] Stefszky M, Ulvila V, Abdallah Z, Silberhorn C, Vainio M (2018). Towards optical frequency-comb generation in continuous-wave-pumped titanium-indiffused lithium-niobate waveguide resonators. Phys. Rev. A (Coll. Park).

[6] Coppin P, Hodgkinson TG (1990). Novel optical frequency comb synthesis using optical feedback. Electron. Lett..

[7] Newbury NR (2011). Searching for applications with a fine-tooth comb. Nat. Photonics.

[8] Ideguchi T, Holzner S, Bernhardt B, Guelachvili G, Picqué N, Hänsch TW (2013). Coherent Raman spectro-imaging with laser frequency combs. Nature.

[9] Coddington I, Swann WC, Nenadovic L, Newbury NR (2009). Rapid and precise absolute distance measurements at long range. Nat. Photonics.

[10] Hinkley N, Sherman JA, Phillips NB, Schioppo M, Lemke ND, Beloy K, Pizzocaro M, Oates CW, Ludlow AD (2013). An atomic clock with 10(-18) instability. Science.

[11] Picqué N, Hänsch TW (2019). Frequency comb spectroscopy. Nat. Photonics.

[12] Schliesser A, Picqué THN, Hänsch TW (2012). Mid-infrared frequency combs. Nat. Photonics.

[13] Atutov SN, Bonazzi F, Calabrese R, Guidi V, Lenisa P, Petruio S, Mariotti E, Moi L (1996). Generation of a frequency comb with a sharp edge of adjustable intensity and frequency. Opt. Commun..

[14] Martín-Mateos P, Jerez B, Acedo P (2015). Dual electro-optic optical frequency combs for multiheterodyne molecular dispersion spectroscopy. Opt. Express.

[15] Martín-Mateos P, Jerez B, Largo-Izquierdo P, Acedo P (2018). Frequency accurate coherent electro-optic dual-comb spectroscopy in real-time. Opt. Express.

[16] Tu H, Xi L, Zhang X, Zhang X, Lin J, Meng W (2013). Analysis of the performance of optical frequency comb based on recirculating frequency shifter influenced by an Er-doped fiber amplifier. Photonics Res..

[17] Durán V, Schnébelin C, Guillet de Chatellus H (2018). Coherent multi-heterodyne spectroscopy using acousto-optic frequency combs. Opt. Express.

[18] Ding Y, Wu B, Shen Y (2021). Acousto-optic frequency shifted comb laser-based micro-Doppler detection for moving target identification. J. Opt. Soc. Am. A.

[19] Billault V, Durán V, Fernández-Pousa CR, Crozatier V, Dolfi D, de Chatellus HG (2021). All-optical coherent pulse compression for dynamic laser ranging using an acousto-optic dual comb. Opt. Express.

[20] Durán V, Chatellus H, Schnebélin C, Nithyanandan K, Djevarhidjian L, Clement J, Fernández-Pousa CR (2019). Optical frequency combs generated by acousto-optic frequency-shifting loops. IEEE Photonics Technol. Lett..

[21] Mantsevich SN, Voloshin AS, Yushkov KB (2019). Optical-frequency-comb generation with collinear acousto-optic diffraction: theory and simulations. Phys. Rev. A (Coll. Park).

[22] Mantsevich SN, Kupreychik MI, Balakshy VI (2020). Possibilities of wide-angle tellurium dioxide acousto-optic cell application for the optical frequency comb generation. Opt. Express.

[23] Mantsevich SN, Yushkov KB, Voloshin AS (2020). Optical frequency combs generation with collinear acousto-optic interaction. Proc. SPIE.

[24] Mantsevich SN, Kostyleva EI (2022). Determination of the paratellurite stiffness constants temperature coefficients by the acousto-optic method. Materialia (Oxford).

[25] Yang Z, Wen M, Wan L, Feng T, Zhou W, Liu D, Zeng S, Yang S, Li Z (2022). Efficient acousto-optic modulation using a microring resonator on a thin-film lithium niobate-chalcogenide hybrid platform. Opt. Lett..

[26] Streifer W, Whinnery JR (1970). Analysis of a dye laser tuned by acoustooptic filter. Appl. Phys. Lett..

[27] Kowalski FV, Hale PD, Shattil SJ (1988). Broadband continuous-wave laser. Opt. Lett..

[28] Littler ICM, Balle S, Bergmann K (1991). Continuous-wave laser without frequency-domain-mode structure: investigation of emission properties and buildup dynamics. J. Opt. Soc. Am. B.

[29] Littler ICM, Balle S, Bergmann K (1992). The cw modeless laser: spectral control, performance data and build-up dynamics. Opt. Commun..

[30] Littler ICM, Eschner JH (1992). The cw modeless laser: model calculations of an active frequency shifted feedback cavity. Opt. Commun..

[31] Ogurtsov VV, Khodakovskyy VM, Yatsenko LP, Shore BW, Bonnet G, Bergmann K (2008). An all-fiber frequency-shifted feedback laser for optical ranging; signal variation with distance. Opt. Commun..

[32] Heidt AM, Burger JP, Maran JN, Traynor N (2007). High power and high energy ultrashort pulse generation with a frequency shifted feedback fiber laser. Opt. Express.

[33] Okhotnikov OG (1998). Multiwavelength picosecond frequency-shifted feedback laser with pulse control by a shaped-gain fiber amplifier. Opt. Lett..

[34] Nikodem MP, Kluźniak E, Abramski K (2009). Wavelength tunability and pulse duration control in frequency shifted feedback Er-doped fiber lasers. Opt. Express.

[35] Vazquez-Zuniga LA, Jeong Y (2013). Study of a mode-locked erbium-doped frequency-shifted-feedback fiber laser incorporating a broad bandpass filter: experimental results. Opt. Commun..

[36] Vazquez-Zuniga LA, Jeong Y (2014). Study of a mode-locked erbium-doped frequency-shifted-feedback fiber laser incorporating a broad bandpass filter: numerical results. Opt. Commun..

[37] Woodward RI, Majewski MR, Jackson SD (2018). Mode-locked dysprosium fiber laser: picosecond pulse generation from 2.97 to 3.30 µm. APL Photonics.

[38] Henderson-Sapir O, Bawden N, Majewski MR, Woodward RI, Ottaway DJ, Jackson SD (2020). Mode-locked and tunable fiber laser at the 3.5 µm band using frequency-shifted feedback. Opt. Lett..

[39] Nikodem M, Abramski K (2010). Controlling the frequency of the frequency-shifted feedback fiber laser using injection-seeding technique. Opt. Commun..

[40] Balle S, Bergmann K (1995). Self-pulsing and instabilities in a unidirectional ring dye laser with intracavity frequency shift. Opt. Commun..

[41] Kim JI, Yatsenko LP, Bergmann K (2022). Ranging with a frequency-shifted feedback laser using frequency-comb driven phase modulation of injected radiation. J. Phys. B.

[42] Nakamura K, Hara T, Yoshida M, Miyahara T, Ito H (2000). Optical frequency domain ranging by a frequency-shifted feedback laser. IEEE J. Quantum Electron..

[43] Yatsenko LP, Shore BW, Bergmann K (2009). Coherence in the output spectrum of frequency shifted feedback lasers. Opt. Commun..

[44] Littler ICM, Keller HM, Gaubatz U, Bregmann K (1991). Velocity control and cooling of an atomic beam using a modeless laser. Z. Phys. D.

[45] Cashen M, Bretin V, Metcalf H (2000). Optical pumping in 4He* with frequency-shifted feedback amplification of light. J. Opt. Soc. Am. B.

[46] Yoshida M, Nakamura K, Ito H (2001). A new method for measurement of group velocity dispersion of optical fibers by using a frequency-shifted feedback fiber laser. IEEE Photonics Technol. Lett..

[47] Guillet de Chatellus H, Jacquin O, Hugon O, Glastre W, Lacot E, Marklof J (2013). Generation of ultrahigh and tunable repetition rates in CW injection-seeded frequency-shifted feedback lasers. Opt. Express.

[48] Yatsenko LP, Shore BW, Bergmann K (2004). Theory of a frequency-shifted feedback laser. Opt. Commun..

[49] Shore KA, Kane DM (1999). Comb generation bandwidth for frequency-shifted feedback semiconductor lasers. IEEE J. Quantum Electron..

[50] Billault V, Crozatier V, Baili G, Morvan L, Dolfi D, Chatellus HG (2020). Dynamic behavior of frequency combs in frequency-shifting loops. J. Opt. Soc. Am. B.

[51] Voloshinov VB (1993). Anisotropic light diffraction on ultrasound in a tellurium dioxide single crystal. Ultrasonics.

[52] Gao Z, Mei T (2022). Spectro-temporal evolution of mode-locked lasing in fiber frequency-shifted feedback laser. Opt. Lett..

[53] Durán V, Djevarhidjian L, Guillet de Chatellus H (2019). Bidirectional frequency-shifting loop for dual-comb spectroscopy. Opt. Lett..

[54] Lucas E, Lihachev G, Bouchand R, Pavlov NG, Raja AS, Karpov M, Gorodetsky M, Kippenberg TJ (2018). Spatial multiplexing of soliton microcombs. Nat. Photonics.

[55] Sun H, Lv H, Wu J, Hu P, Fu H, Yang H, Yang R, Ding X (2021). Subringwavelength multidimensional multiplexing for quad-comb generation from an integrated dual-ring mode-locked laser. Proc. SPIE.

[56] Li T, Zhao X, Chen J, Li Q, Xie S, Zheng Z (2019). Tri-comb and quad-combgeneration based on a multi-dimensional multiplexed mode-locked laser. J. Lightwave Technol..

[57] Yang J, Liu J, Li T, Hu J, Wang J, Wu Y, Xie S, Zhao X, Zheng Z (2022). Dynamic spectroscopic characterization for fast spectral variations based on dual asynchronous undersampling with triple optical frequency combs. Opt. Lasers Eng..

[58] Lomsadze B, Smith BC, Cundiff ST (2018). Tri-comb spectroscopy. Nat. Photonics.

[59] Coddington I, Newbury N, Swann W (2016). Dual-comb spectroscopy. Optica.

